# Therapeutic Applications of Botulinum Neurotoxins in Veterinary Medicine

**DOI:** 10.3390/vetsci10070460

**Published:** 2023-07-13

**Authors:** Lauretta Turin, Marina Michela Piccione, Fabio Crosa, Paola Dall’Ara, Joel Filipe, Laura Zarucco

**Affiliations:** 1 Department of Veterinary Medicine and Animal Sciences (DIVAS), University of Milan, Via dell’Università 6, 26900 Lodi, LO, Italy; marina.piccione@istituto-besta.it (M.M.P.); paola.dallara@unimi.it (P.D.); joel.soares@unimi.it (J.F.); 2 Department of Veterinary Sciences (DSV), University of Turin, Largo Paolo Braccini 2, 10095 Grugliasco, TO, Italy; fabio.crosa88@gmail.com (F.C.); laura.zarucco@unito.it (L.Z.)

**Keywords:** *Clostridium botulinum*, botulism, neurotoxin, BoNT, therapeutic, antibodies, horse, dog, cat, farm animals

## Abstract

**Simple Summary:**

Botulinum neurotoxins have been considered for therapeutic effects in different animal species and for several conditions. However, a review of the reports and the identification of the research priorities in this field is still lacking. This study provides an overview of the current knowledge on the characteristics and the mechanism of action of the available botulinum neurotoxin formulations applied in veterinary medicine, identifying critical issues and research gaps. Overall, the literature largely supports the beneficial activity of such toxins to manage pain and to treat a variety of dystonias and highlights the need for additional research.

**Abstract:**

Botulinum neurotoxins (BoNTs) are emerging as multipurpose therapeutic compounds for the treatment of several different syndromes involving peripheral and central nervous systems, and muscular and musculoskeletal disorders both in human and veterinary medicine. Therefore, the study of BoNTs is rapidly developing and identifying newly produced BoNT variants. Efforts should be made to clarify the biological and pharmacological characteristics of these novel BoNTs as well as the natural ones. The high potential of BoNTs as a therapeutic compound for medical syndromes lies in its ability to reach a specific cell type while bypassing other cells, thus having mild or no side effects. In this paper the recent developments in BoNTs are reviewed with the aim of analyzing the current knowledge on BoNTs’ biological mechanisms of action, immunogenicity, formulations, and therapeutic applications in the veterinary field, highlighting advantages and drawbacks and identifying the gaps to be filled in order to address research priorities.

## 1. Introduction

Botulinum neurotoxins (BoNTs) are proteins synthesized and secreted by neurotoxigenic strains of anaerobic and sporogenous bacteria belonging to the genus *Clostridium*. The BoNTs are conventionally classified into seven serological types, designated with alphabetical letters from A to G (BoNT-A through BoNT-G) [1] plus two recently discovered serotypes (BoNT-H previously considered A/F chimera and BoNT-X) [2]. Only serotypes A, B, E and F (possibly G and H) target humans causing poisoning, whilst serotypes C and D are responsible for botulism in animals, mainly cattle, sheep, horses, birds, and fishes [3]. Neurotoxin producer *C. botulinum* strains are historically distinguished into four groups (I, II, III and IV) according to physiologic and metabolic characteristics [4]. Nevertheless, *C. botulinum* is not the only BoNT-producer, since other bacteria belonging to the same genus are BoNT-producers, such as *C. butyricum* (BoNT-E), *C. baratii* (BoNT-F), and *C. argentinense* (BoNT-G) [4]. More recently, advanced molecular technologies enabled the discovery of novel BoNTs, which were clustered as subtypes within the existing serological types and designated with the letter of the serotype accompanied by a number (e.g., for serotype A: BoNT-A1, BoNT-A2, BoNT-An) [5]. BoNT subtypes vary in amino acid sequence and therefore may differ in antigenic properties. Moreover, chimeric BoNTs were discovered as a result of the recombination of *bont* genes and designated with both the letters of the originating serotypes (e.g., BoNT/CD, BoNT/FA). In this way, the number of subtypes has more than doubled and continues to grow. The increasing number of BoNT variants has a relevant biological significance and may be explained by environmental pressure on neurotoxigenic strains of *Clostridia* responsible for animal botulism. Novel BoNTs can also be designed and produced via recombinant technology. Since even small differences in the amino acid sequence can significantly change the activity and the toxicity, improved and/or different BoNTs are expected to be obtained in the near future in a natural or recombinant way.

The toxin induces muscle paralysis, a disease known as “botulism” in infected, untreated vertebrates, by inhibiting the release of the acetylcholine neurotransmitter at the cholinergic neuromuscular junctions. In the most serious conditions, it may reach the limbs, ending in systemic paralysis [6]. The paralysis of respiratory muscles causes respiratory arrest and finally death. Botulism may take one of three main presentations. The first one is a foodborne disease, which occurs after ingestion of toxin-contaminated food or drinks, the second presentation is wound botulism, which arises after the entry of the toxin through an open wound, and the third one is infant botulism, a less frequent form that results after gastrointestinal infection of the infant immature gut by toxin-producing clostridia, which release the toxin systemically causing a flaccid paralysis known as “floppy baby syndrome”. In the absence of treatment, victims are immediately placed in intensive care for a long time, usually under mechanical ventilation, up till the paralysis wears off. In the case of infant botulism, antibiotic administration may be required to treat other dangerous bacterial complications [7]. Despite the high toxicity, numerous preparations based on BoNTs are commercially available and extensively used for therapeutic purposes to improve conditions affecting neuromuscular or autonomic neuronal transmission. Botulinum toxin can also be used for cosmetic purposes.

## 2. Structure of Botulinum Neurotoxin

**Figure 1:**
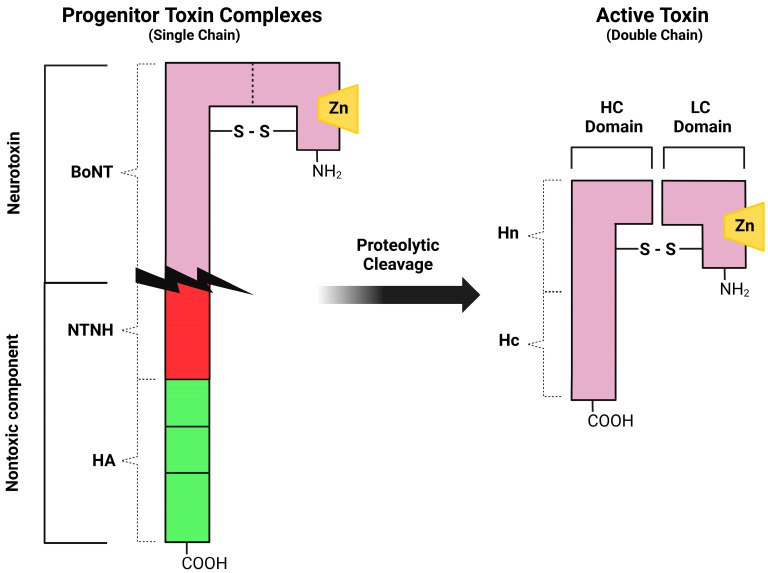
Schematic representation of botulinum neurotoxin type A. The toxin is generated as an inactive single chain protein; then it is activated by proteolytic cleavage to a double-chain form (HC, heavy chain; LC, light chain). Three primary domains, the receptor binding domain (Hc), the translocation domain (Hn) and the catalytic domain (LC) are present in the neurotoxin.

The genes (*bont*) encoding for BoNT serotype A, B, E and F are carried by the bacterial chromosome, the ones producing serotypes C and D by a phage genome, and the gene for BoNT-G by a plasmid [1]. The *bont* genes encode for 150-kDa proteins that enfold in a three-domain arrangement adjacent to a cluster of genes (*ntnha*) coding for accessory proteins NTNHA (non-toxic non-hemagglutinin), which play the triple role of preserving the core neurotoxin from low gastric pH upon oral introduction, promoting gastrointestinal absorption, and shielding the BoNT molecule from proteolytic and other chemical attacks while passing through the protease-rich gastrointestinal tract [8]. These functions are crucial since the most common portal of entry of the ingested BoNTs into the body is the gut. The *bont* and *ntnha* genes are located next to the *ha* genes that code for neurotoxin-associated proteins with hemagglutination properties in some bacterial strains, while in other strains they are proximal to genes *orfX*, whose functions are still under investigation [9]. HA is involved in the disruption of the intestinal epithelial cell barrier, allowing botulinum toxin to enter the body [10]. The key feature of the HA cluster proteins is the abundance of carbohydrate-binding sites that can bind to the mucus layer and to the apical membrane of polarized intestinal epithelial cells, or other cells of the gut wall, by which BoNTs infiltrate the lymphatic and blood circulation [5]. BoNTs, along with their accessory non-toxic proteins, generate large composite molecules named Progenitor Toxin Complexes (PTCs). Such complexes, although not involving covalent binding, are stable at acidic pH [7], but at neutral or slightly basic pH conditions (as in the inner intestinal polarized epithelial monolayer and the intramuscular fluids) the ‘‘pH sensor’’ amino acids of the NTNHA molecule trigger a conformational modification and cause the release of the core neurotoxin [11]. Although different in amino acidic composition and immunogenicity, all BoNT serotypes display a similar molecular architecture. They are inactive when produced as a single-chain (150 kDa) polypeptide but then they are cleaved by proteases to generate the mature (and pharmacologically active) toxin, consisting of a light chain (LC, 50 kDa) joined by a single disulfide bridge to a heavy chain (HC, 100 kDa) comprising the binding domain at the carboxy-terminus (Hc, 50 kDa) and the translocation domain at the amino-terminus (Hn, 50 kDa) (Figure 1) [12].

## 3. Mechanism of Action

**Figure 2:**
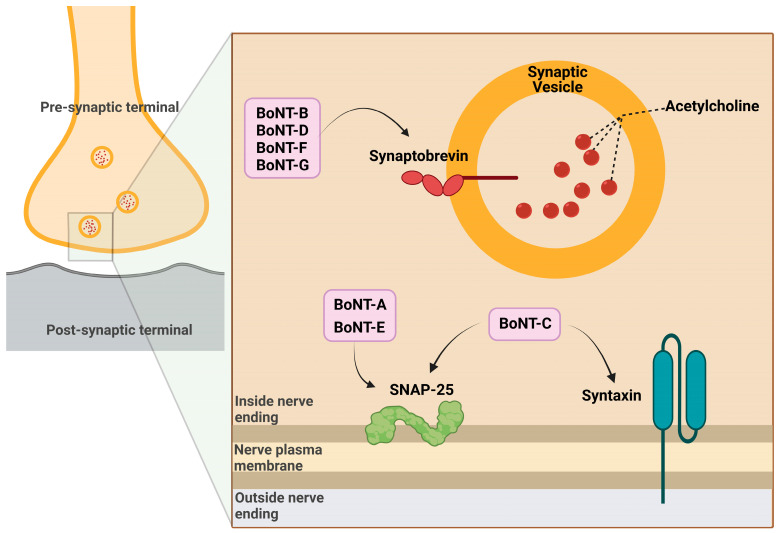
Mechanism of action of botulinum toxin in the pre-synaptic terminal.

BoNTs, after reaching the lymphatic and blood circulation (through gut absorption, inhalation, or injection), quickly reach the perineuronal fluid compartment (without crossing the blood–brain barrier) and bind to presynaptic neurons [6]. BoNT mainly works as a significant inhibitor of acetylcholine release by the presynaptic neurons. The normal release of neurotransmitters in brain synapses involves the fusion of synaptic vesicles with cell plasma membranes and requires a protein complex called SNARE (soluble N-ethylmaleimide sensitive factor attachment protein receptor) or SNAP receptor [13]. After binding with the carboxy-terminus of the heavy chain to the presynaptic nerve membrane, BoNTs are endocytosed into synaptic vesicles with the involvement of adenosine triphosphatase (ATPase) proton pumps [14]. The vesicular acidic pH triggers a conformational switch of BoNT, which favors the HN-mediated translocation of the light chain of BoNT across the endosomal membrane into the cytoplasm [15]. The reducing cytosolic environment enhances disulfide bond cleavage, allowing the catalytic LC zinc protease domain of BoNT to freely act, cleaving the substrate represented by different polypeptide portions of the SNARE complex depending on the BoNT serotype [8,16]. In particular, LC zinc proteases of BoNT-A and BoNT-E breakdown SNAP25, proteases of BoNT-B, BoNT-D, BoNT-F and BoNT-G hydrolyze VAMP/synaptobrevin (vesicle-associated membrane protein) and BoNT-C degrades either SNAP25 (synaptosomal associated membrane protein of 25 kDa) or syntaxin [14] (Figure 2). After the degradation of SNARE, the fusion of acetylcholine vesicles with the plasma membrane is impeded and finally the neuromuscular transmission is blocked [15], causing flaccid muscle paralysis and possibly death [7]. Neurospecificity, binding affinity and catalytic activity, which characterize the biological mechanism of action of BoNTs, are the bases for their toxicity on the one hand and their successful pharmacological and therapeutic applications on the other. For these potentialities, it is necessary to deepen the knowledge of the mechanisms that regulate BoNTs activity.

## 4. Immunogenicity and Formulations

The BoNT core neurotoxin, as well as the linked accessory proteins are potential immunogens [17]. This feature represents a major disadvantage for their therapeutic application, especially when requiring multiple administrations. Therefore, strategies have been or are still being developed to prepare formulations with minimized immunogenicity of the biological compound.

Accessory proteins do not play any therapeutic function, but at neutral pH they dissociate from the neurotoxin and act as adjuvants [18] promoting both the synthesis of neutralizing antibodies against the active BoNT [19] and the expression of inflammatory cytokines [20]. They also bind to different cell types other than neurons, inducing undesired immune-mediated responses [21]. Consequently, a minimal presence of accessory proteins in BoNT formulations is desirable.

Differing from the accessory proteins, the BoNT core neurotoxin exhibits low immunogenicity, particularly BoNT-A, which is the serotype currently mostly used as a drug [22]. Three BoNT-A and one BoNT-B preparations have been authorized for some time by the FDA (Food and Drug Administration) and EMA (European Medicines Agency) for therapeutic utilization, each having a different potency: onabotulinumtoxin-A (onaBoNT-A or Botox^®^, Allergan Pharmaceuticals, Dublin, Ireland; former Oculinum^®^, Oculinum Inc., Irvine, CA, USA), abobotulinumtoxin-A (aboBoNT-A or Dysport^®^, Ipsen Biopharm Ltd., Paris, France, Galderma Ltd., Zug, Switzerland), incobotulinumtoxin-A (incoBoNT-A or Xeomin^®^, Merz Pharmaceuticals GmbH, Frankfurt, Germany) and rimabotulinumtoxin-B (rimaBoNT-B or Myoblock^®^, Solstice Neurosciences, Llc., Louisville, KY, USA); a fourth BoNT-A more recently developed, daxibotulinumtoxin-A (daxiBoNT-A, Revance Therapeutics, Pleasanton, CA, USA) [23] has been also approved. These formulations are conventionally distinguished into first- and second-generation, and differ in components, such as excipients and accessory protein content [8,14,24].

- First-generation BoNT-A formulations:
  onaBoNT-A (first authorized by the FDA in 1989) consists of 0.73 ng of core neurotoxin protein (a mix of active protein and inactive/denatured toxoid) complexed with about 4.3 ng of accessory proteins [25];
  aboBoNT-A contains less core neurotoxin (0.65 ng) than onaBoNT-A complexed with about 3.7 ng of accessory and other clostridia-derived proteins such as flagellin [26];
  rimaBoNT-B consists of core neurotoxin complexed with accessory proteins [27].

- Second-generation BoNT-A formulations:
  incoBoNT-A contains the therapeutic neurotoxin only (0.44 ng), free of any accessory protein or any other bacterial-derived molecule [28];
  daxiBoNT-A lacks bacterial accessory proteins, but the composition has not been disclosed for therapeutic applications [29].

The second-generation products, compared with the first-generation ones, are lacking the accessory proteins and display significantly lower immunogenicity along with high clinical efficacy, better safety profile, and the possibility to be stored at room temperature. The achievement of low immunogenicity is a clinically relevant feature to avoid treatment resistance due to the action of neutralizing antibodies at the time of secondary administration or further cumulative dosages. Nevertheless, immunogenicity is just one of the potential risk factors linked to BoNT treatment. Other parameters need to be carefully considered, such as cumulative dose, injection site, schedule of injections, duration of treatment, protein load, patient status, and choice of BoNT formulation. More insightful knowledge of the mechanisms involved in the development of the immune response against BoNTs is undoubtedly needed to further improve the formulations and to prevent or treat BoNT-related immune resistance by minimizing immune reactions and maximizing the effect.

## 5. Therapeutic Applications in Veterinary Medicine

BoNT molecules are characterized by multiple beneficial pharmacological properties, which make them particularly versatile drugs. They combine potent neurospecificity with reversibility of their effect (temporary striated muscle paralysis) and limited diffusion when locally injected. These features explain why the utilization of the purified form of botulinum toxin from the anaerobic bacteria *Clostridium botulinum* is now well-established both in human and veterinary clinical practice. BoNT is used to treat numerous chronic conditions including neuromuscular syndromes such as dystonia, dysphagia, blepharospasm, autonomic syndromes such as hyperfunction of parasympathetic nerves, gastrointestinal (achalasia, gustatory sweating syndrome, anal fissure), secretory (hyperhidrosis) and urogenital (overactive bladder, benign prostatic hypertrophy) disorders. It is also used as a chronic pain killer for neuropathic and non-neuropathic disorders [16,30,31]. Many other applications are being investigated.

### 5.1. Horse

When compared to other animal species including humans, horses are very susceptible to BoNT, which can be poisoning and often deadly (foodborne or wound botulism) [32]. Nevertheless, encouraging results were obtained using BoNT-A and -B for therapy in this species. Few pilot studies were conducted on horses demonstrating the efficacy and safety of local injections of botulinum neurotoxin to cure stringhalt, laminitis, acute synovitis, lameness and anal pressure.

- Stringhalt (equine reflex hypertonia) is a spasticity condition consisting of recurrent hyperflexion of the tarsus potentially due to several reasons, among which are neurogenic causes linked to dysfunctional or over-reacting upper motor neurons. Being limited in the success of conventional remedies for stringhalt, BoNT-A (Botox) injections in the *extensor digitorum longus*, *extensor digitorum lateralis* and *lateral vastus* muscles were tested, and results showed diminished spastic movements and less frequent hypermetric steps as early as 2 days post-inoculation, in absence of toxicity signs and adverse side effects. The only partial success obtained with this trial (the spasticity was not completely abolished) shows that dosage and type of muscles to be injected are critical variables that need to be fine-tuned [33].
- Botulinum neurotoxin type A was also evaluated as a treatment for horse laminitis, an extremely painful condition caused by inflammation of the laminae bonding the hoof wall to the distal phalanx in the hoof. Laminitis may turn into a more severe condition (founder and/or sinker) when the animal weight and locomotion forces applied by the deep digital flexor (DDF) muscle on the distal phalanx (coffin bone), cause the failure of the lamellar attachment between the coffin bone and the hoof capsule. In this condition BoNT-A, causing paralysis of the DDF muscle, diminishes the shearing forces and improves the sequelae of laminitis. Horses with different degrees of laminitis treated with BoNT-A (Botox) in the muscle belly of the deep digital flexor muscle presented no worsening of the disease and Obel scores improved by a few grades [34]. Further studies have confirmed the potential of BoNT-A (Botox) in the treatment of horses with laminitis by assessing the effectiveness of the intramuscular injection of neurotoxin on the reduction in DDF muscle activity, increased range of motion of the metacarpus and carpus and force distribution underneath the hooves in healthy, adult sport horses. No significant changes were detected in the toe-heel force distribution, neither in gait alterations upon walking [35,36].
- A horse model of lameness associated with acute synovitis was utilized to determine the result of intra-articular injection of BoNT-A. Joint pain is one of the most frequent lameness-triggering factors in horses. After induction of acute synovitis in horses with normal carpi and BoNT-A (Botox) injection into the middle carpal joint, only a few of them showed onset of lameness. These results indicate that BoNT has a pain-relieving effect besides a neuromuscular blocking one. Intra-articular BoNT-A administration can alleviate lameness in horses with acute synovitis without any adverse effect [37]. A similar study was recently published to demonstrate that the injection of BoNT-A into the carpal joint of healthy horses does not cause any negative effect on synovial and clinical parameters and is therefore safe for use in horses [38].
- Chronic forelimb lameness is often the result of pain from the navicular bone and the soft tissue of the podotrochlear apparatus (PA) following degenerative disease in horses. Since the PA is rich in unmyelinated nerve fibers, possibly transmitting foot pain to the spinal cord, horses with severe PA radiographic and tomographic abnormalities and lameness showed short-term clinical improvement after intrabursal BoNT-B injection. BoNT-B, by binding the synovial nociceptor fibers of the PA, can inhibit the release of other SNARE-dependent neuropeptides responsible for the transmission of foot pain to the spinal cord. Horses intrabursally inoculated with BoNT-B displayed improved lameness for more than 14 days and no adverse effects. However, a total lameness-free condition was not achieved. Multiple injections or higher BoNT-B doses/concentrations/volumes may be needed to optimize the analgesic effect before the clinical application can be recommended in horses [39].
- Botulinum toxin type B has also been shown to reduce anal sphincter tone in horses. Foaling is often the cause of perineal lacerations in mares that may dehisce after surgical repair due to the high pressure exerted by the accumulation of stool in the rectum. Local injection of BoNT-B into the external anal sphincter of mares before surgical intervention to repair perineal lacerations showed a reduction in incisional dehiscence due to induction of transient relaxation of the anus and lower anal tone. The maximal efficacy was achieved in the first 15 days post-inoculation and disappeared after about 6 months. Although not fully successful (only transient relaxation), this treatment shows good potential for improvement [40].

Overall, the results obtained so far in horses, even if only partially positive, encourage further investigations with larger animal cohorts and confirm that horses are more sensitive to BoNT than other animal species and humans. Therefore, it should be emphasized that careful optimization of doses, together with the timing of administration and guidelines on injection sites, are crucial aspects for the successful outcome of the therapy.

### 5.2. Dog

According to one of the first available references, dogs are more resistant than humans to all types of botulinum toxin, with a greater resistance to type C followed by type A [41]. Therefore, the onset of effects after BoNT inoculation is expected to be similar to that of the natural toxin, but the efficacy is expected to be lower and of shorter duration at an equivalent dose. In dogs, BoNT-A represents a reversible, safe, and effective alternative to more invasive procedures, and has been used to treat otolaryngologist-head and -neck problems, as well as gastrointestinal and urinary disorders, osteoarthritis and pain, atrial fibrillation, myoclonus, myokymia and neuromyotonia, ocular complications and asthma.

- The first study known in dogs aimed to assess the potential benefit of BoNT in canine patients with otolaryngological head and neck disorders and was performed more than three decades ago. In this study, the BoNT-A (Oculinum) injection into the cricothyroid muscle of a dog with bilateral abductor vocal cord paralysis caused muscle paralysis and lowered the tension of the vocal cords, allowing it to move laterally and therefore improving the airflow safely [42]. A second study published soon after by the same research group, confirmed the efficacy of multiple BoNT-A (Oculinum) injections into the laryngeal muscle to solve airway stenosis caused by bilateral abductor vocal cord paralysis without morbidity, permanent damage to laryngeal muscles, dysphagia, or mortality [43]. Additionally, canine laryngeal hyperadduction disorders, such as spasmodic dysphonia, were temporarily treated with BoNT-A (Botox) injections into the thyroarytenoid muscle with or without radiofrequency-induced thermotherapy without severe side effects [44,45]. Finally, excessive salivation (ptyalism) in dogs was significantly reduced by BoNT-A and -D (injections into submandibular glands, where they could act in the neuroglandular junction by blocking acetylcholine secretion. The parasympathetic postganglionic neurons innervating the canine submandibular glands are sensitive to the anticholinergic effect of BoNTs (and particularly BoNT-D) without side effects [46].
- Some dysfunctions of the canine gastrointestinal apparatus have been successfully treated with BoNT. Functional obstruction of the lower esophageal sphincter (achalasia-like syndrome) is due to the loss of inhibitory myenteric neurons leading to a lack of relaxation after the pharyngeal swallow and altered esophageal motility (megaesophagus), with poor prognosis. This dysfunction was recently improved (although only for about 40 days) with BoNT-A (Botox) injections at eight sites around the esophagogastric junction coupled, or not, to surgical myotomy, without long-term complications [47]. A canine model to study the relaxing effect of BoNT-A (Botox) injected into the ampulla of Vater, under endoscopy guidance, in alternative to biliary stenting or sphincterotomy, showed a significant reduction in pressure gradient between the common bile duct and the duodenum in case of dysfunctions of the sphincter of Oddi, such as biliary fistulae or leaks. The effect that paralyzes the muscular valve and therefore achieves relaxation of the sphincter started within 24 h post-treatment and lasted at least 14 days, displaying low invasiveness and complexity with no complications [48,49]. Finally, a case of delayed gastric emptying, a frequent dog dysfunction caused by anatomic (foreign bodies or masses), functional (inflammation, infection, or idiopathic) or emotional (stress) drainage block or by reduced gastric motility, was successfully solved with laparoscopic BoNT-A (Botox) injections into the pylorus [50]. In all these cases, the toxin exerted its blockage activity of muscle/sphincter contraction with minimal or no adverse effects, justifying further studies to optimize its use.
- Since dogs can naturally present benign prostatic hyperplasia (BPH, 80% of intact 5-year-old or older males), they represent a perfect target and a good animal model for the application of botulinum neurotoxic treatment. The results of three separate studies performed by transperitoneal BoNT-A (Botox or Dysport) injection into each lobe of the prostate of BPH dogs demonstrated a prostate size and firmness reduction and a glandular atrophy and apoptosis increase for more than 3 months in the absence of any complication or side effect or a negative impact on the semen quality [51,52,53]. These effects may be due to both relaxation of the prostatic muscle and alteration of growth factors expression due to the toxin [54]. BoNT proved to be effective also in a canine bladder reconstruction model in which gastrocystoplasty with demucosalized patches coated by engineered urothelial mucosa together with BoNT-A (Botox) injection had a clinical potential in bladder reconstruction in patients with non-compliant bladder [55]. Finally, urinary incontinence (UI) was successfully controlled in 70% of bitches with the application of a dozen injections of BoNT-A (Botox) into different sites of the bladder wall submucosa, with sustained benefit for about 5 months in the absence of side effects [56]. Therefore, all of these studies provided robust information to support the efficacy of BoNT. However, they highlight some critical points that need to be addressed, such as the mechanism of BoNT interference with the urothelial functions, the volume and site of administration and the duration of effect.
- Attempts were made to use BoNT for the most frequent joint disease in dogs, which is osteoarthritis (OA), a painful life-quality impacting condition involving cartilage deterioration and bone remodeling. It was hypothesized that, by inhibiting the liberation of neuropeptides at the nociceptive nerve endings, BoNT-A may work as an analgesic drug. In a preliminary study, performed on dogs with osteoarthritis secondary to hip dysplasia, injections of BoNT-A (Dysport) failed to show significant improvement probably because of insufficient dose or the OA severity of the enrolled dogs [57]. Another study confirmed the lack of significant benefit after intra-articular injection of BoNT-A (Botox) and started to clarify the antinociceptive mechanism of action of the toxin, finding that it is not bound to molecules that normally transmit signals in arthritis such as substance P and prostaglandin E2 [58]. Nevertheless, another study from the same research group had demonstrated that BoNT-A (Botox) inoculation into joints of osteoarthritic dogs significantly lowered the pain, with efficacy peak at 12 weeks post-injections and without remarkable systemic or local side effects [59]. Similarly, a pilot study on a limited number of dogs with mild to severe osteoarthritis showed augmented ground reaction forces in dogs after injection of BoNT-A (Botox) [60]. Overall, all these studies showed that the optimal therapeutic BoNT dosages still need to be optimized and the inclusion criteria for the animals should also be carefully defined because osteoarthritis is a complex disease involving numerous factors whose mechanisms are largely unknown and for which efficacious therapies are continuously searched for. Noteworthy, a recently published study demonstrated that intra-articular injections of BoNT-A (Botox) in healthy dogs do not induce adverse (cytological, clinical, or histopathological) effects and can therefore be considered safe for use in dogs [61].
- Other than for osteoarthritic pain, the analgesic effect of BoNT-A (Dysport) was tested in post-operative bilateral radical canine mastectomy and showed usefulness as an adjuvant antinociceptive agent to control pain, effective when injected prior to surgery [62]. By inhibiting the release of neuropeptides at the nociceptive nerve endings, BoNT-A lowers directly peripheral, and indirectly central sensitivity, providing analgesia.
- Dog heart, like the human heart, has three main epicardial fat pads abundantly and asymmetrically innervated with ganglionated plexi of the autonomic nervous system. Since atrial fibrillation depends on electrophysiological parasympathetic and sympathetic stimuli, botulinum neurotoxin-induced denervation was investigated in a canine model as an atrial fibrillation suppression strategy. The results of two different studies demonstrated that the injection of onabotulinumtoxin-A (Botox) into the two, or abobotulinumtoxin-A (Dysport) into the four major atrial ganglionated plexi significantly lowered atrial fibrillation inducibility for three weeks and three months, respectively [63,64]. These short- and long-term results demonstrate the basics for an attractive non-invasive, transitory, but still effective procedure. Another study performed in dogs to determine the potential effect of injections of BoNT-A (Botox) into the left stellate ganglion in post-myocardial infarction, demonstrated the inhibited function of the sympathetic nervous system, improved cardiac remodeling and function and prevention of ventricular arrhythmias, ultimately showing beneficial cardioprotective effects of the toxin [65].
- BoNT-A (Botox) showed a positive effect on dog myoclonus, a particularly debilitating disease with involuntary, irregular twitching of a part, an entire, or a group of muscles that can hamper walking, eating, and dog health in general. Since myoclonus is produced by an aberrant firing pattern of lower motor neurons, injections of toxin into the most severely affected muscles (nearby the motor end plate) resulted in improved walking and other motor functions without severe adverse effects [66]. Similarly, myokymia and neuromyotonia secondary to radiation therapy were effectively treated in dogs by BoNT-A (Botox) injections (twice in 24 h) into the affected muscles, safely and without side effects [67]. The drawback of this therapy is the duration of the effect, which lasted up to 6 and 3 months, respectively, and therefore requires repeated treatments.
- Canine ocular complications can occur following conjunctivitis, keratitis, entropion, or foreign bodies. Protective ptosis may be temporarily necessary in some cases to protect the cornea and allow healing of the ocular disease, to avoid surgery. From this perspective, transcutaneous injections of BoNT-A in the anterior levator palpebral superioris muscle allowed effective covering of the cornea for up to 3 weeks in a canine experimental study [68]. Local subcutaneous injections of BoNT-A (Dysport) were also effective in the treatment of primary dog blepharospasm, an abnormal, uncontrolled contraction of the eyelid muscles [69]. In this case, injections of botulinum toxin A into the orbicularis oculi muscle were repeated every 4 months without adverse signs for over 3 years. Studies with a larger cohort of patients would be desirable to consolidate these promising outcomes.
- An experimental study to reduce bronchial hyperreactivity in dogs tested the effect of BoNT-A submucosal injections into the caudal lobe bronchus. Local injections of BoNT-A resulted in a reduction of about 60% of the bronchial hyper-responsiveness, chemically induced to mimic asthma, in a canine model for up to 6 months [70].

All studies on canine patients demonstrate that BoNT-A treatments bring remarkable clinical improvement to diseases lacking efficacious treatments and characterized by high morbidity and poor quality of life. More insights into dose-dependent effect, inoculation sites and schedule would be needed to prolong the duration of treatment efficacy and to better understand the BoNT-A mechanism of action.

### 5.3. Cat

Although less documented than dogs and horses, cats have also been treated with botulinum neurotoxins for severe contractures, and as animal models to study ocular motility disorders.

- In a case of acquired limb deformity with severe muscle contracture, successful application of BoNT-A (Botox) in a juvenile cat was reported as an alternative to limb amputation. Botox injections into the triceps and flexor carpi and digit muscles, relieved pain, and decreased muscle spasticity, facilitating physiotherapy and the application of a brace [71]. Partial success was also previously reported for BoNT-A (Botox) injections into the gastrocnemius muscle in an elderly cat presenting with tarsal arthrogryposis [72]. The minimal improvement obtained in this case could be explained by the lower dose of BoNT-A used and the congenital nature of the deformity with severe joint contractures.
- Since cats are frontal-eyed species characterized by postnatal maturation of the palisade endings of the extraocular muscles (axonal specializations that combine motor and sensory features), they represent a good model to study the functional relationship between sensory and motor features in the palisade endings. An initial study, a few decades ago, showed that only one injection of botulinum neurotoxin into the ocular retrobulbar orbit was sufficient to cause complete or partial reproducible paralysis of the ocular musculature lasting up to one month [73]. This methodology has shown promise for further studies of ocular motility. A few years later, injections of high doses of BoNT-A into the lateral rectus muscle were shown to impair not only the electrical activity and therefore eye movement, but also the firing rate of the abducens motoneurons, while low doses only caused muscle paralysis of the lateral rectus [74]. This finding supported the safe use of BoNT-A at low doses to induce therapeutic relaxation of spastic eye muscles, avoiding functional perturbations of the motoneurons and therefore of the central nervous system. Nonetheless, a study investigating possible ultrastructural changes due to injections of BoNT-A (Botox) into the feline extraocular muscles demonstrated ultrastructural changes (mild myelin separation at the proximal part of the myotendinous nerve endings and an augmented number of neurofilaments in the myelinated and unmyelinated nerve fibers axons) impacting the proprioceptive function of the extraocular muscle, which should be taken into consideration [75]. More insights into the BoNT dose effect and mechanism of action were subsequently clarified for Botox [76], and finally, it was demonstrated that the eye immobilization but not the visual deprivation affects palisade ending development. Moreover, during the development time, the palisade endings are subject to perturbations [77]. These studies have a crucial impact on designing therapies for oculomotor dystonias.

Based on the outcomes of the few reported feline studies and the established and extensive application of BoNT in human medicine, Botulinum-based neurotoxic treatments warrant future investigations as a targeted therapy or therapeutic aid.

### 5.4. Farm Animals (Pig, Sheep)

Most studies performed with botulinum neurotoxin in farm animals involved pigs. Only one study is reported on sheep.

#### 5.4.1. Pig

Pigs, although naturally resistant to botulism, can harbor botulinum toxin types B and C in their intestine [78]. However, they respond to direct administration of BoNT and therefore they are frequently used as animal models for translational studies, targeting masticatory musculature and gastrointestinal and urinary systems.

- A first original study was published a decade ago and was aimed at testing the effect of intramuscular injection of BoNT-A (Botox) into masseter muscle of pigs, demonstrating relevant alterations of the fiber composition and myosin expression (mRNA), therefore mainly resulting in an impact on the structure rather than on the functionality (very bland local paresis) [79]. Another study comparing the effects of BoNT-A (Botox) and BoNT-B (Myobloc) injections in the masseter muscle of minipigs showed no paresis or atrophy with BoNT-A, but atrophy and paresis with BoNT-B [78].

The doses of BoNT administered to pigs are comparable to those used for humans allowing useful information for human medicine.

- Newborn piglets have been utilized in pilot studies to investigate the effect of intramural esophageal injections of BoNT-A on the relaxation and elongation of the esophagus as an animal model for pediatric esophageal atresia, a congenital defect, which requires esophageal anastomosis. BoNT-A (Xeomin) injections into the muscular layer of the esophageal wall followed 1 hour later by surgical removal of the entire esophagus and an in vitro stretch-tension elongation test, showed a significant esophageal elongation in treated vs. control animals [80]. When the time laps between BoNT-A injections and the stress-tension test doubled to two hours, the elongation increased significantly [81].

Despite these encouraging preliminary results, issues persist with this animal model because of differences between newborn humans and piglets’ esophagi. In particular, the human esophagus is more fragile and elastic than that of piglets. This may strongly impact the dose-effect curve and the timing of the effect. Furthermore, the effect of BoNT-A on the esophagus is currently controversial, as a recent publication of a piglet study showed no differences in the mechanical and histological characteristics of the esophagus in BoNT-A-injected piglets compared to controls [82].

Sphincters of the porcine gastrointestinal apparatus, being anatomically similar to those of humans, have been targeted as a model to enlighten the potential effect of botulinum neurotoxin.

- Different from the upper esophageal sphincter, which is composed of skeletal muscles (on which BoNT has proven to be efficacious), the lower esophageal sphincter is composed of smooth muscle. In the piglet model, the local (intrasphinteric) effect of BoNT-A (Oculinum) injections into this gastrointestinal smooth muscle was investigated and showed a relevant (60%) muscle tone reduction in the absence of side effects [83]. This study proved for the first time that BoNT has potential applications in the treatment of gastrointestinal sphincter disorders such as achalasia and Oddi dysfunction [83]. However, mucosal inflammation of the esophagus and fibrosis of the lower esophageal sphincter in adult pigs injected with BoNT-A (Botox) was further highlighted [84].
- BoNT-A has also been tested in the pig model to assess the effect on internal anal sphincter dysfunctions such as achalasia and fissure. Injections of BoNT-A (Botox) into the internal anal sphincter, at the anal skin and rectal mucosa interface of piglets, induced a significant decrease in the internal anal sphincter tonicity, safely, reversibly, and with no side effects. Hypertonicity is responsible for persistent obstructive symptoms, which may occur in children, for which piglets represent a suitable model [85]. The effect of BoNT-A (Botox) was also tested in the adult porcine model and showed diminished myogenic tone and reduced contractions in response to sympathetic nerve stimulation when injected into the internal anal sphincter or the intersphincteric space [86]. These direct or indirect effects of BoNT on sympathetic nerves are similar to the ones detected with Botox for urethra [87,88,89] and may therefore serve as a model to treat chronic anal fissure.
- The effect of BoNT-A (Botox) on the morphological and chemical phenotype of the autonomic nerve fibers, which innervate the urinary bladder wall, was tested by injecting it into the urinary bladder wall of pigs. As a result, altered nerve fiber distribution and frequency were observed, along with changes in the expression pattern of adrenergic and cholinergic traits, ultimately indicating high plasticity and adaptability of urinary bladder wall neurons [87]. The same research group clarified the changes in the expression patterns of neurochemical molecules by cholinergic nerves (sympathetic ganglia) after multiple injections of Botox into the urinary bladder wall and demonstrated that the therapeutic effect of the toxin on this organ is partly due to sympathetic ganglia through their altered expression of neuropeptides [88,89].

BoNT-A was also evaluated in the pig model to possibly diminish the tone of the ureteral detrusor muscle and therefore facilitate stone passage through the ureterovesical junction and finally expulsion. Periureteral injections into three sites of the detrusor muscle of BoNT-A (Botox) determined relaxation of the muscle and faster stone passage [90].

Lately, the application of BoNT has gained relevance in the treatment of urinary tract syndromes, therefore the porcine model, highly similar to the human one, is suitable to study such therapeutics.

Pigs are excellent models for translational studies to humans because they may contribute relevant information on the use of BoNT, for instance on various orofacial movement pathologies such as masseteric hypertrophy, temporomandibular joint disorders, musculoskeletal pain, and bruxism. Finally, a recent study conducted on minipigs demonstrated that BoNT-A injected into the panniculus carnosus shortened the expansion time of the myocutaneous flap, reducing resistance, accelerating inflation, increasing the expansion area, and minimizing contraction [91]. This could hold great potential for reconstructive surgical procedures.

#### 5.4.2. Sheep

In a study on subacute atrial fibrillation, BoNT-A (Botox) injections into right and left atrial fat pads, pulmonary veins, and the anterior surface of the right and left ventricles (two injections at each site) lowered the susceptibility to atrial fibrillation induction as early as 7 days after injection and for 3 months, in absence of vagal stimulation and without side effects [92]. This study, albeit with the limitations of a small number of animals, and a one-week follow-up, showed the efficacy of BoNT-A in reducing the vulnerability of the atrial myocardium to fibrillation.

## 6. Conclusions and Perspectives

Botulinum neurotoxins, by interfering with the release of acetylcholine at neuromuscular junctions, have emerged as beneficial therapeutic compounds for a broad panel of syndromes including muscle, musculoskeletal and neuropathic disorders. While clinical evidence supports BoNT treatments, unanswered questions persist, for example about the functionality and potential immunogenicity of the formulations. Therefore, further research is needed to elucidate the mechanism and duration of action of the toxin and how it triggers the host immune response. This can then lead to the design and development of new and improved engineered BoNT biomolecules, ideally selective for each, to better address different clinical needs, including additional pathologies. For example, by modifying the heavy chain it is possible to bind to different target cells, where the toxin itself can enter or act as a vector, allowing the entry of other therapeutic proteins into specific cells. In this way, a wider applicability for BoNTs can be achieved. Furthermore, additional preclinical studies would be essential to define patient selection, indication, dose, delivery system and safety. Animals, in addition to representing useful study models, are themselves targets of the effect of BoNTs on disease symptoms. However, success in animal models may fail to translate to humans, suggesting the need for caution.

The evaluation of therapeutic value must therefore consider not only the relief and remission of symptoms but also the recurrence rate.

Finally, advances in knowledge have made the potent botulinum toxin a safe, effective, minimally invasive, and reversible curative remedy with the potential to further enhance the beneficial impact with the application of BoNT in vaccines or for the production of new constructs [93].

## Data Availability

Not applicable.

## References

[1] Smith T.J., Hill K.K., Raphael B.H. (2015). Historical and current perspectives on *Clostridium botulinum* diversity. Res. Microbiol..

[2] Zhang S., Masuyer G., Zhang J., Shen Y., Henriksson L., Miyashita S.I., Martínez-Carranza M., Dong M., Stenmark P. (2017). Identification and characterization of a novel botulinum neurotoxin. Nat. Commun..

[3] Dong M., Stenmark P. (2021). The structure and classification of botulinum toxins. Botulinum Toxin Therapy.

[4] Collins M.D., East A.K. (1997). Phylogeny and taxonomy of the food-borne pathogen *Clostridium botulinum* and its neurotoxins. J. Appl. Microbiol..

[5] Pirazzini M., Rossetto O., Eleopra R., Montecucco C. (2017). Botulinum neurotoxins: Biology, pharmacology, and toxicology. Pharmacol. Rev..

[6] Simpson L. (2013). The life history of a botulinum toxin molecule. Toxicon.

[7] Masuyer G., Chaddock J.A., Foster K.A., Acharya K.R. (2014). Engineered botulinum neurotoxins as new therapeutics. Annu. Rev. Pharmacol. Toxicol..

[8] Carr W.W., Jain N., Sublett J.W. (2021). Immunogenicity of botulinum toxin formulations: Potential therapeutic implications. Adv. Ther..

[9] Gao L., Lam K.H., Liu S., Przykopanski A., Lübke J., Qi R., Krüger M., Nowakowska M.B., Selby K., Douillard F.P. (2023). Crystal structures of OrfX1, OrfX2 and the OrfX1-OrfX3 complex from the orfX gene cluster of botulinum neurotoxin E1. FEBS Lett..

[10] Matsumura T., Sugawara Y., Yutani M., Amatsu S., Yagita H., Kohda T., Fukuoka S., Nakamura Y., Fukuda S., Hase K. (2015). Botulinum toxin A complex exploits intestinal M cells to enter the host and exert neurotoxicity. Nat. Commun..

[11] Gu S., Rumpel S., Zhou J., Strotmeier J., Bigalke H., Perry K., Shoemaker C.B., Rummel A., Jin R. (2012). Botulinum neurotoxin is shielded by NTNHA in an interlocked complex. Science.

[12] Turton K., Chaddock J.A., Acharya K.R. (2002). Botulinum and tetanus neurotoxins: Structure, function and therapeutic utility. Trends Biochem. Sci..

[13] Südhof T.C., Rothman J.E. (2009). Membrane fusion: Grappling with SNARE and SM proteins. Science.

[14] Jankovic J. (2017). Botulinum toxin: State of the art. Mov. Disord..

[15] Montal M. (2010). Botulinum neurotoxin: A marvel of protein design. Annu. Rev. Biochem..

[16] Kostrzewa R.M., Kostrzewa R.A., Kostrzewa J.P. (2015). Botulinum neurotoxin: Progress in negating its neurotoxicity; and in extending its therapeutic utility via molecular engineering. Peptides.

[17] Critchfield J. (2002). Considering the immune response to botulinum toxin. Clin. J. Pain.

[18] Kukreja R., Chang T.W., Cai S., Lindo P., Riding S., Zhou Y., Ravichandran E., Singh B.R. (2009). Immunological characterization of the subunits of type A botulinum neurotoxin and different components of its associated proteins. Toxicon.

[19] Bryant A.M., Cai S., Singh B.R. (2013). Comparative immunochemical characteristics of botulinum neurotoxin type A and its associated proteins. Toxicon.

[20] Lee J.C., Yokota K., Arimitsu H., Hwang H.J., Sakaguchi Y., Cui J., Takeshi K., Watanabe T., Ohyama T., Oguma K. (2005). Production of anti-neurotoxin antibody is enhanced by two subcomponents, HA1 and HA3b, of *Clostridium botulinum* type B 16S toxin-haemagglutinin. Microbiology.

[21] Wang L., Sun Y., Yang W., Lindo P., Singh B.R. (2014). Type A botulinum neurotoxin complex proteins differentially modulate host response of neuronal cells. Toxicon.

[22] Bellows S., Jankovic J. (2019). Immunogenicity associated with botulinum toxin treatment. Toxins.

[23] Carruthers J., Solish N., Humphrey S., Rosen N., Muhn C., Bertucci V., Swift A., Metelitsa A., Rubio R.G., Waugh J. (2017). Injectable DaxibotulinumtoxinA for the treatment of glabellar lines: A phase 2, randomized, dose-ranging, double-blind, multicenter comparison with onabotulinumtoxinA and placebo. Dermatol. Surg..

[24] Fonfria E., Maignel J., Lezmi S., Martin V., Splevins A., Shubber S., Kalinichev M., Foster K., Picaut P., Krupp J. (2018). The expanding therapeutic utility of botulinum neurotoxins. Toxins.

[25] Allergan (2020). BOTOX (Onabotulinumtoxina) for Injection, for Intramuscular, Intradetrusor, or Intradermal Use Prescribing Information. https://media.allergan.com/actavis/actavis/media/allergan-pdf-documents/product-prescribing/20190620-BOTOX-100-and-200-Units-v3-0USPI1145-v2-0MG1145.pdf.

[26] Ipsen (2018). DYSPORT (Abobotulinumtoxina) for Injection, for Intramuscular Use Prescribing Information. https://www.ipsen.com/websites/Ipsen_Online/wp-content/uploads/sites/9/2019/01/21084019/Dysport_Full_Prescribing_Information.pdf.

[27] Solstice Neurosciences (2000). MYOBLOCK (Rimabotulinumtoxinb) for Injection, for Intramuscular Use Prescribing Information. https://www.myobloc.com/files/MYOBLOC_PI.pdf.

[28] Merz (2019). XEOMIN (Incobotulinumtoxina) for Injection, for Intramuscular or Intraglandular Use Prescribing Information. https://www.xeominaesthetic.com/wp-content/uploads/2019/05/XEOMIN-Full-Prescribing-Information-including-MedGuide.pdf.

[29] Solish N., Carruthers J., Kaufman J., Rubio R.G., Gross T.M., Gallagher C.J. (2021). Overview of DaxibotulinumtoxinA for injection: A novel formulation of botulinum toxin type A. Drugs.

[30] Kostrzewa R.M., Segura-Aguilar J. (2007). Botulinum neurotoxin: Evolution from poison to research tool--onto medicinal therapeutic and future pharmaceutical panacea. Neurotox. Res..

[31] Cohen S.P. (2015). Botulinum toxin type B for chronic pain: Panacea or snake oil? The need for more and better preclinical studies. Anesth. Analg..

[32] Whitlock R.H., Buckley C. (1997). Botulism. Vet. Clin. North Am. Equine Pract..

[33] Wijnberg I.D., Schrama S.E., Elgersma A.E., Maree J.T., de Cocq P., Back W. (2009). Quantification of surface EMG signals to monitor the effect of a Botox treatment in six healthy ponies and two horses with stringhalt: Preliminary study. Equine Vet. J..

[34] Carter D.W., Renfroe J.B. (2009). A novel approach to the treatment and prevention of laminitis: Botulinum toxin type A for the treatment of laminitis. J. Equine Vet. Sci..

[35] Hardeman L.C., van der Meij B.R., Oosterlinck M., Veraa S., van der Kolk J.H., Wijnberg I.D., Back W. (2013). Effect of *Clostridium botulinum* toxin type A injections into the deep digital flexor muscle on the range of motion of the metacarpus and carpus, and the force distribution underneath the hooves, of sound horses at the walk. Vet. J..

[36] Wijnberg I.D., Hardeman L.C., van der Meij B.R., Veraa S., Back W., van der Kolk J.H. (2013). The effect of *Clostridium botulinum* toxin type A injections on motor unit activity of the deep digital flexor muscle in healthy sound Royal Dutch sport horses. Vet. J..

[37] DePuy T., Howard R., Keegan K., Wilson D., Kramer J., Cook J.L., Childers M.K. (2007). Effects of intra-articular botulinum toxin type A in an equine model of acute synovitis: A pilot study. Am. J. Phys. Med. Rehabil..

[38] Júnior A.A.B., Paz L.B., Frank M.I., Engelmann A.M., Krause A., Côrte F.D. (2022). Safety and synovial inflammatory response after intra-articular injection of botulinum toxin Type A in healthy horses. J. Equine Vet. Sci..

[39] Gutierrez-Nibeyro S.D., Santos M.P., White N.A., Brown J.A., Adams M.N., McKnight A.L., Schaeffer D.J. (2014). Effects of intrabursal administration of botulinum toxin type B on lameness in horses with degenerative injury to the podotrochlear apparatus. Am. J. Vet. Res..

[40] Adam-Castrillo D., White N.A., Donaldson L.L., Furr M.O. (2004). Effects of injection of botulinum toxin type B into the external anal sphincter on anal pressure of horses. Am. J. Vet. Res..

[41] Graham R., Eriksen S. (1922). Experimental botulism in dogs. J. Infect. Dis..

[42] Cohen S.R., Thompson J.W. (1987). Use of botulinum toxin to lateralize true vocal cords: A biochemical method to relieve bilateral abductor vocal cord paralysis. Ann. Otol. Rhinol. Laryngol..

[43] Cohen S.R., Thompson J.W., Camilon F.S. (1989). Botulinum toxin for relief of bilateral abductor paralysis of the larynx: Histologic study in an animal model. Ann. Otol. Rhinol. Laryngol..

[44] Sercarz J.A., Berke G.S., Ming Y., Rothschiller J., Graves M.C. (1992). Bilateral thyroarytenoid denervation: A new treatment for laryngeal hyperadduction disorders studied in the canine. Otolaryngol. Head Neck Surg..

[45] Desai S.C., Park A.M., Chernock R.D., Paniello R.C. (2016). Minithyrotomy with radiofrequency-induced thermotherapy for the treatment of adductor spasmodic dysphonia. Laryngoscope.

[46] Shaari C.M., Wu B.L., Biller H.F., Chuang S.K., Sanders I. (1998). Botulinum toxin decreases salivation from canine submandibular glands. Otolaryngol. Head Neck Surg..

[47] Grobman M.E., Hutcheson K.D., Lever T.E., Mann F.A., Reinero C.R. (2019). Mechanical dilation, botulinum toxin A injection, and surgical myotomy with fundoplication for treatment of lower esophageal sphincter achalasia-like syndrome in dogs. J. Vet. Intern. Med..

[48] Marks J.M., Bower A.L., Goormastic M., Malycky J.L., Ponsky J.L. (2001). A comparison of common bile duct pressures after botulinum toxin injection into the sphincter of Oddi versus biliary stenting in a canine model. Am. J. Surg..

[49] Brodsky J.A., Marks J.M., Malm J.A., Bower A., Ponsky J.L. (2002). Sphincter of Oddi injection with botulinum toxin is as effective as endobiliary stent in resolving cystic duct leaks in a canine model. Gastrointest. Endosc..

[50] Rinaldi M.L., Fransson B.A., Barry S.L. (2014). Botulinum toxin A as a treatment for delayed gastric emptying in a dog. Can. Vet. J..

[51] Chuang Y.C., Tu C.H., Huang C.C., Lin H.J., Chiang P.H., Yoshimura N., Chancellor M.B. (2006). Intraprostatic injection of botulinum toxin type-A relieves bladder outlet obstruction in human and induces prostate apoptosis in dogs. BMC Urol..

[52] Lin A.T., Yang A.H., Chen K.K. (2007). Effects of botulinum toxin A on the contractile function of dog prostate. Eur. Urol..

[53] Mostachio G.Q., Apparício M., Motheo T.F., Alves A.E., Vicente W.R. (2012). Intra-prostatic injection of botulinum toxin type A in treatment of dogs with spontaneous benign prostatic hyperplasia. Anim. Reprod. Sci..

[54] Cruz F. (2014). Targets for botulinum toxin in the lower urinary tract. Neurourol. Urodyn..

[55] Zhang Y., Liu G., Kropp B.P. (2012). Re-epithelialization of demucosalized stomach patch with tissue-engineered urothelial mucosa combined with Botox A in bladder augmentation. BJU Int..

[56] Lew S., Majewski M., Radziszewski P., Kuleta Z. (2010). Therapeutic efficacy of botulinum toxin in the treatment of urinary incontinence in female dogs. Acta Vet. Hung..

[57] Nicácio G.M., Luna S.P.L., Cavaleti P., Cassu R.N. (2019). Intra-articular botulinum toxin A (BoNT/A) for pain management in dogs with osteoarthritis secondary to hip dysplasia: A randomized controlled clinical trial. J. Vet. Med. Sci..

[58] Heikkilä H.M., Hielm-Björkman A.K., Innes J.F., Laitinen-Vapaavuori O.M. (2017). The effect of intra-articular botulinum toxin A on substance P, prostaglandin E_2_, and tumor necrosis factor alpha in the canine osteoarthritic joint. BMC Vet. Res..

[59] Heikkilä H.M., Hielm-Björkman A.K., Morelius M., Larsen S., Honkavaara J., Innes J.F., Laitinen-Vapaavuori O.M. (2014). Intra-articular botulinum toxin A for the treatment of osteoarthritic joint pain in dogs: A randomized, double-blinded, placebo-controlled clinical trial. Vet. J..

[60] Hadley H.S., Wheeler J.L., Petersen S.W. (2010). Effects of intra-articular botulinum toxin type A (Botox) in dogs with chronic osteoarthritis. Vet. Comp. Orthop. Traumatol..

[61] Heikkilä H.M., Jokinen T.S., Syrjä P., Junnila J., Hielm-Björkman A., Laitinen-Vapaavuori O. (2018). Assessing adverse effects of intra-articular botulinum toxin A in healthy Beagle dogs: A placebo-controlled, blinded, randomized trial. PLoS ONE.

[62] Vilhegas S., Cassu R.N., Barbero R.C., Crociolli G.C., Rocha T.L., Gomes D.R. (2015). Botulinum toxin type A as an adjunct in postoperative pain management in dogs undergoing radical mastectomy. Vet. Rec..

[63] Oh S., Choi E.K., Zhang Y., Mazgalev T.N. (2011). Botulinum toxin injection in epicardial autonomic ganglia temporarily suppresses vagally mediated atrial fibrillation. Circ. Arrhythm. Electrophysiol..

[64] Lo L.W., Chang H.Y., Scherlag B.J., Lin Y.J., Chou Y.H., Lin W.L., Chen S.A., Po S.S. (2016). Temporary suppression of cardiac ganglionated plexi leads to long-term suppression of atrial fibrillation: Evidence of early autonomic intervention to break the vicious cycle of “AF Begets AF”. J. Am. Heart Assoc..

[65] Zhang S., Wang M., Jiao L., Liu C., Chen H., Zhou L., Wang Y., Wang Y., Liu Z., Liu Z. (2022). Ultrasound-guided injection of botulinum toxin type A blocks cardiac sympathetic ganglion to improve cardiac remodeling in a large animal model of chronic myocardial infarction. Heart Rhythm.

[66] Schubert T., Clemmons R., Miles S., Draper W. (2013). The use of botulinum toxin for the treatment of generalized myoclonus in a dog. J. Am. Anim. Hosp. Assoc..

[67] Rogatko C.P., Glass E.N., Kent M., Hammond J.J., de Lahunta A. (2016). Use of botulinum toxin type A for the treatment of radiation therapy-induced myokymia and neuromyotonia in a dog. J. Am. Vet. Med. Assoc..

[68] Bittencourt M.K., de Vasconcellos J.P., Bittencourt M.D., Malagó R., Bacellar M. (2013). Evaluation of the efficacy and safety of botulinum toxin type A to induce temporary ptosis in dogs. J. Ocul. Pharmacol. Ther..

[69] Meyer-Lindenberg A., Wohlfarth K.M., Switzer E.N. (2003). The use of botulinum toxin A for treatment of possible essential blepharospasm in a dog. Aust. Vet. J..

[70] Al-Halfawy A., Gomaa N.E., Refaat A., Wissa M., Wahidi M.M. (2012). Endobronchial injection of botulinum toxin for the reduction of bronchial hyperreactivity induced by methacholine inhalation in dogs. J. Bronchol. Interv. Pulmonol..

[71] McGeachan R.I., Schwarz T., Gunn-Moore D.A., Marioni-Henry K. (2020). Botulinum toxin type A for the treatment of muscle contractures secondary to acute spinal cord injury in a young cat. JFMS Open Rep..

[72] Bright S.R., Girling S.L., O’Neill T., Innes J.F. (2007). Partial tarsal arthrodesis and botulinum toxin A injection for correction of tarsal arthrogryposis in a cat. J. Small Anim. Pract..

[73] Zimm J., Yanik G.M., Evans L., Marchese A. (1991). Reduction of cat eye movements using retrobulbar botulinum toxin. J. Ocul. Pharmacol..

[74] Moreno-López B., de la Cruz R.R., Pastor A.M., Delgado-García J.M. (1994). Botulinum neurotoxin alters the discharge characteristics of abducens motoneurons in the alert cat. J. Neurophysiol..

[75] Suh Y.W., Uhm C.S., Cho Y.A. (2010). Ultrastructural changes in myotendinous nerve endings induced by injection of botulinum toxin into the extraocular muscle. Graefes Arch. Clin. Exp. Ophthalmol..

[76] Dimitrova D.M., Shall M.S., Goldberg S.J. (2002). Short-term effects of botulinum toxin on the lateral rectus muscle of the cat. Exp. Brain Res..

[77] Carrero-Rojas G., Calvo P.M., Lischka T., Streicher J., de la Cruz R.R., Pastor A.M., Blumer R. (2022). Eye movements but not vision drive the development of palisade endings. Investig. Ophthalmol. Vis. Sci..

[78] Liu Z.J., Rafferty K.L., Ye W., Herring S.W. (2015). Differential response of pig masseter to botulinum neurotoxin serotypes a and b. Muscle Nerve.

[79] Gedrange T., Gredes T., Spassov A., Mai R., Kuhn D.U., Dominiak M., Kunert-Keil C. (2013). Histological changes and changes in the myosin mRNA content of the porcine masticatory muscles after masseter treatment with botulinum toxin A. Clin. Oral Investig..

[80] Larsen H.F., Jensen T.S., Rasmussen L., Ellebæk M., Qvist N. (2013). Intramural injection with botulinum toxin significantly elongates the pig esophagus. J. Pediatr. Surg..

[81] Dibbern C.B., Rose M., Ellebæk M.B., Qvist N. (2017). The effect of intramural botulinum toxin injections on the elongation of the piglet oesophagus is time dependent. Eur. J. Pediatr. Surg..

[82] Rose M., Clarke P., Pike A.H., Zvara P., Schrøder H.D., Hejboel E.K., Qvist N., Ellebæk M.B. (2022). Endoscopic injections of botulinum toxin type A in the piglet esophagus is safe and feasible but did not result in any significant structural changes 3 days after injection. Eur. J. Pediatr. Surg..

[83] Pasricha P.J., Ravich W.J., Kalloo A.N. (1993). Effects of intrasphincteric botulinum toxin on the lower esophageal sphincter in piglets. Gastroenterology.

[84] Richardson W.S., Willis G.W., Smith J.W. (2003). Evaluation of scar formation after botulinum toxin injection or forced balloon dilation to the lower esophageal sphincter. Surg. Endosc..

[85] Langer J.C., Birnbaum E.E., Schmidt R.E. (1997). Histology and function of the internal anal sphincter after injection of botulinum toxin. J. Surg. Res..

[86] Jones O.M., Moore J.A., Brading A.F., Mortensen N.J. (2003). Botulinum toxin injection inhibits myogenic tone and sympathetic nerve function in the porcine internal anal sphincter. Colorectal Dis..

[87] Lepiarczyk E., Bossowska A., Kaleczyc J., Majewski M. (2011). The influence of botulinum toxin type A (BTX) on the immunohistochemical characteristics of noradrenergic and cholinergic nerve fibers supplying the porcine urinary bladder wall. Pol. J. Vet. Sci..

[88] Bossowska A., Majewski M. (2012). Botulinum toxin type A-induced changes in the chemical coding of dorsal root ganglion neurons supplying the porcine urinary bladder. Pol. J. Vet. Sci..

[89] Lepiarczyk E., Bossowska A., Majewski M. (2015). Changes in chemical coding of sympathetic chain ganglia (SChG) neurons supplying porcine urinary bladder after botulinum toxin (BTX) treatment. Cell Tissue Res..

[90] Streeper N.M., Nakada S.Y., Wertheim M.L., Best S.L. (2016). Preliminary evidence suggests periureteral botulinum toxin type A injection improves ureteral stone passage in the porcine model. J. Endourol..

[91] Chenwang D., Shiwei B., Dashan Y., Qiang L., Bin C., Muxin Z., Pengcheng L., Senkai L. (2009). Application of botulinum toxin type A in myocutaneous flap expansion. Plast. Reconstr. Surg..

[92] Nazeri A., Ganapathy A.V., Massumi A., Massumi M., Tuzun E., Stainback R., Segura A.M., Elayda M.A., Razavi M. (2017). Effect of botulinum toxin on inducibility and maintenance of atrial fibrillation in ovine myocardial tissue. Pacing Clin. Electrophysiol..

[93] Leese C., Christmas C., Mészáros J., Ward S., Maiaru M., Hunt S.P., Davletov B. (2023). New botulinum neurotoxin constructs for treatment of chronic pain. Life Sci. Alliance.

